# Evidence of the Presence of Low Pathogenic Avian Influenza A Viruses in Wild Waterfowl in 2018 in South Africa

**DOI:** 10.3390/pathogens8040163

**Published:** 2019-09-25

**Authors:** Marjolein J. Poen, Ron A. M. Fouchier, Richard J. Webby, Robert G. Webster, Mohamed E. El Zowalaty

**Affiliations:** 1 Department of Viroscience, Erasmus Medical Center, Dr. Molewaterplein 50, 3015GE Rotterdam, The Netherlands; m.poen@erasmusmc.nl (M.J.P.); r.fouchier@erasmusmc.nl (R.A.M.F.); 2 Division of Virology, Department of Infectious Diseases, Center of Excellence for Influenza Research and Surveillance (CEIRS), St. Jude Children’s Research Hospital, 262 Danny Thomas Place, Memphis, TN 38105, USA; Richard.webby@stjude.org (R.J.W.); Robert.webster@stjude.org (R.G.W.); 3 Virology and Microbiology Research Group, Department of Pharmacy, City University College of Ajman, Sheikh Amaar Road, Al Tallah 2, P.O. Box 18484 Ajman, United Arab Emirates

**Keywords:** avian influenza, epidemiology, influenza A virus, migratory waterfowl, real-time RT-PCR, South Africa, surveillance, wild birds, zoonosis

## Abstract

Avian influenza viruses are pathogens of global concern to both animal and human health. Wild birds are the natural reservoir of avian influenza viruses and facilitate virus transport over large distances. Surprisingly, limited research has been performed to determine avian influenza host species and virus dynamics in wild birds on the African continent, including South Africa. This study described the first wild bird surveillance efforts for influenza A viruses in KwaZulu-Natal Province in South Africa after the 2017/2018 outbreak with highly pathogenic avian influenza virus H5N8 in poultry. A total of 550 swab samples from 278 migratory waterfowl were tested using real-time RT-PCR methods. Two samples (0.7%) were positive for avian influenza virus based on the matrix gene real-time RT-PCR but were negative for the hemagglutinin subtypes H5 and H7. Unfortunately, no sequence information or viable virus could be retrieved from the samples. This study shows that avian influenza viruses are present in the South African wild bird population, emphasizing the need for more extensive surveillance studies to determine the South African avian influenza gene pool and relevant local host species.

## 1. Introduction

Avian influenza viruses (AIVs) continue to pose a threat to both animal and human health worldwide. Influenza A viruses belong to the family *Orthomyxoviridae* [1] and are classified into 18 HA and 11 NA subtypes, of which 16 HA (H1–H16) and 9 NA (N1–N9) subtypes circulate in avian species; H17N10 [2] and H18N11 [3] influenza A subtypes were detected in bats captured from South America using molecular methods. Wild birds of the orders *Anseriformes* (like ducks, geese and swans) and *Charadriiformes* (like gulls, waders and terns) are the main natural reservoir of influenza A virus subtypes [4,5]. AIVs of the H5 and H7 subtypes are further classified into low pathogenic avian influenza (LPAI) and highly pathogenic avian influenza (HPAI) viruses based on molecular markers and mortality rates in experimentally intravenously inoculated chickens [6]. In contrast to LPAI viruses, HPAI viruses cause high morbidity and mortality in poultry, but cause a spectrum of disease in wild birds. In addition, HPAI viruses have been reported to infect humans, resulting in high case mortality rates [7]. In 2005, HPAI viruses of the H5N1 subtype were detected in Asia, Europe, the Middle East and Africa, including South Africa, causing devastating economical losses due to numerous infections in both poultry and wild birds [7,8]. More recently, three other HPAI H5 subtypes emerged: H5N8 in 2014/15, H5N8 in 2016/17 and H5N6 in 2017/18. These HPAI viruses were most likely dispersed by wild migratory birds that travel long distances during their spring and autumn migration [9].

South Africa is a subtropical country located in the south of the African continent in the West Asian–East African and the East Atlantic migratory bird flyways. Due to the wide range of climatic types and habitats, South Africa has a wide variety of wild birds, both resident and migratory. Avian influenza surveillance efforts in South Africa have been very limited and mainly restricted to poultry species, including commercially kept ostriches. In June 2017, the HPAI H5N8 virus was first detected in domestic, captive and wild bird species in South Africa [10]. Due to the lack of surveillance activities prior to this detection, it is unknown when and how the virus first entered the country. However, the close proximity of commercial poultry farms to rivers and wetlands harboring high numbers of wild migratory waterfowl creates an ideal environment for wild-domestic transmission of AIVs. This study reports the results of active surveillance activities in KwaZulu-Natal Province in South Africa, a location previously marked as a high-risk area [11]. The aim of this project was to determine the temporal and spatial distribution of AIVs in waterfowl in this region.

## 2. Materials and Methods

### 2.1. Ethical Approvals

Bird catch, ring and release permits were obtained from Ezemvelo KwaZulu-Natal Wildlife Authority, Cascades, Pietermaritzburg, South Africa and KwaZulu-Natal Department of Agriculture and Rural Development, Veterinary Services, Cascades, Pietermaritzburg, South Africa. All procedures were conducted in full compliance with Section 20 of Animal Diseases Act 35 of 1984 and were approved by the South African Department of Agriculture, Forestry and Fisheries (Section 20 approval Reference. 12/11/1/5 granted to Prof. Mohamed Ezzat El Zowalaty). In addition, the research was conducted in compliance with the South African Council for Non-proliferation of Weapons of Mass Destruction (Ref. number NPC 018/416 granted to Prof. Mohamed Ezzat El Zowalaty). Ethical approvals were obtained from the Animal Research Ethics Committee of the University of KwaZulu-Natal (Reference AREC 071/017).

### 2.2. Sample Collection

**Figure 1:**
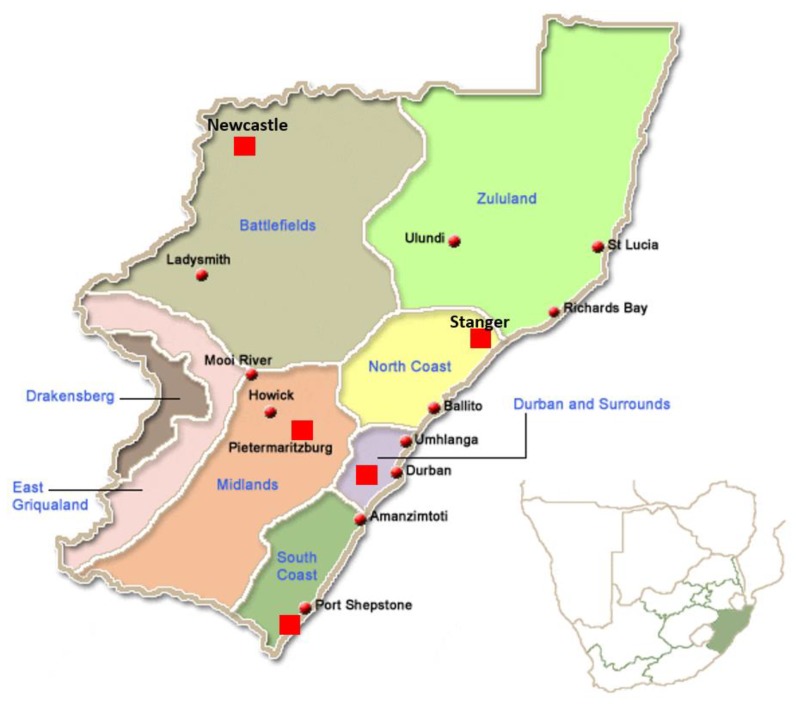
Geographic map showing sampling locations in this study.

Samples were collected from migratory waterfowl of the orders *Anseriformes* and *Charadriformes* captured from four different areas in the KwaZulu-Natal Province between February and December 2018 from different areas in Stanger (North Coast), Durban, Darvill Waste Water in Pietermaritzburg, and Newcastle Ponds (Figure 1). Birds were caught using mist netting and night-lighting.

Samples were collected using sterile swabs, transferred into 2 mL viral transport medium as described previously [12], and samples were stored at −80 °C until shipped and processed.

### 2.3. Virus Detection, Isolation, Characterisation and Sequencing

The samples were tested at the Erasmus Medical Center for the presence of avian influenza A viruses using matrix gene specific, and H5 and H7 HA gene specific real-time RT-PCR (RRT-PCR) assays with a cut-off for negative samples set at a cycle threshold (Ct) value of 40 on the basis of the findings for multiple amplification curves as previously described [13]. Only positive samples were inoculated in two 11-day-old embryonated chicken eggs for 3 days (no blind passaging) and sequenced using Sanger (Thermo Fisher Scientific, Waltham, MA, USA) and MinION (Oxford Nanopore Technologies, Oxford, United Kingdom) techniques as previously described [14,15].

## 3. Results

**Figure 2:**
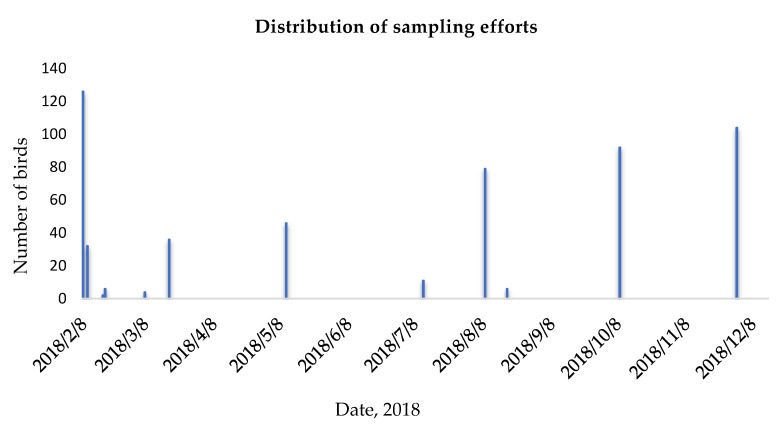
Distribution of sample collection activities in number of birds (y-axis) in time (x-axis) during AI surveillance effort from wild birds in South Africa between February and December, 2018.

A total of 550 cloacal and oropharyngeal swabs were collected from 278 wild birds from 19 different species representing five families (Table 1, Figure 2).

The majority of birds were caught alive without any signs of disease. In addition, swabs were obtained from four Egyptian geese showing signs of disease (diarrhoea and lethargy) and two Egyptian geese found dead, all in the Durban area. The samples were collected from different sampling areas in KwaZulu-Natal Province (Figure 1). The majority of samples (n = 500) were obtained from birds captured in Durban, mainly Egyptian geese (n = 425), the rest of the samples were collected from species other than Egyptian geese from Stanger (n = 8), Pietermaritzburg (n = 4), and the Newcastle area (n = 32); we were unable to determine the source location of 6 samples.

Two samples, a cloacal and an oropharyngeal swab sample, from two healthy juvenile Egyptian geese captured in the Durban area on 9 October 2018 tested positive for the presence of avian influenza A virus (2/278-0.7%), but were negative for the presence of the H5 and H7 HA gene. Both samples contained low quantities of viral RNA (Ct values were 34.88 and 34.7) and could not be successfully cultured nor sequenced. Due to the initial high Ct value and the low sensitivity of HA-subtype specific RRT-PCR assays [16], no further subtyping was performed.

## 4. Discussion

Surveillance and timely reporting are essential to increase our understanding of avian influenza epidemiology in wild birds and other hosts. This study reports the first AI surveillance efforts in wild waterfowl in the KwaZulu-Natal Province in South Africa, and showed evidence of a low incidence (0.7%) of AI infections in the wild bird population in 2018. Despite the fact that the two positive samples could not be subtyped, diagnostically excluding the H5 and H7 subtypes indicated that both were likely LPAI viruses of other subtypes. Wild bird surveillance efforts in South Africa are very limited [17,18,19,20], despite the fact that South Africa may be at a constant risk of HPAI introduction because of its wild bird flyway connection to and overlap with areas where HPAI viruses have/may become endemic, like Egypt [21], and infections in South African poultry have been described [19,22,23,24]. The factors, virologic, ornithologic, or epidemiologic that influence the dissemination of influenza viruses across regions by wild birds are poorly understood. In addition, some areas, like KwaZulu-Natal Province, have previously been marked as AIV high-risk areas based on wild bird movement [11]. The current status of avian influenza virus circulation in the South African wild bird population is unknown. To the authors’ knowledge, the most recently published reports describing the detection of AIVs in living wild birds in South Africa (H1N8 and H4N2 in Egyptian geese) date back to 2007–2009 [19,20], apart from published detections of HPAI H5 viruses in outbreak-related wild bird screening [25]. However, based on sequence information deposited into public databases like Influenza Research Database (https://www.fludb.org), GenBank (https://www.ncbi.nlm.nih.gov/genbank) and Global Initiative on Sharing All Influenza Data (GISAID) (https://www.gisaid.org), the most recent detections of LPAI viruses in wild birds date back to 2012/2013. AIVs that were detected in South Africa thus far belong to Eurasian lineage viruses, although the genetic distance is quite large indicating intermediate viruses might have circulated in an unknown reservoir [19]. This is supported by other studies that have suggested that direct contact between Eurasian birds and local South African birds is a less likely route of avian influenza virus introduction into South Africa [26]. Compared to previous LPAI incidence data from South Africa (1.1%) [26], this study shows a similar incidence of 0.7%. However, because we targeted high-risk species in this study (e.g., Egyptian geese [19]), a higher incidence might be expected. Moreover, European AIV incidences are generally higher with clear seasonal peaks in late summer and autumn [27]. Possible explanations may include the difference in the ecology of AIV in the southern hemisphere compared to the northern hemisphere due to the milder winter, arid environment, the absence of migratory Palearctic duck species, and the flexible movement strategies of African wild bird populations that complicate the determination of (locally) involved hosts and the optimal timing of surveillance [26,28]. To address these issues, a broader range of species in representative numbers should be monitored regularly over a longer period of time.

Much of our knowledge about AIV dynamics is based on surveillance and epidemiological investigations in wild migratory birds. AIV surveillance in wild birds will help the understanding of the dynamics of AIV spread within and across continents and will, therefore, contribute to the development of successful control measures and strategies to manage and reduce the impact of (HPAI) virus outbreaks. In addition, continued surveillance efforts will help monitor the viral gene pool and trace the origins of influenza A viruses in South African wild birds and poultry and should remain a research priority as part of global surveillance programs.

## Figures and Tables

**Table 1:** Detection of avian influenza virus in avian samples obtained from 19 species of waterfowl captured in South Africa in 2018.

Bird Species	No. of Birds Captured	No. of Cloacal Samples Tested	No. of Oropharyngeal Samples Tested	No. AIV Positive by RRT-PCR	Bird Capture Location
African Jacana *(Actophilornis africanus)*	1	1	1	0	Stanger
Bahama Pintail Duck *(Anas bahamensis)*	3	3	3	0	Durban
Blacksmith Lapwing *(Vanellus armatus)*	5	5	5	0	Newcastle (n = 3) Pietermaritzburg (n = 2)
Carolina Wood Duck *(Aix sponsa)*	2	2	2	0	Durban
Common Sandpiper *(Actitis hypoleucos)*	1	1	1	0	Newcastle
Egyptian Geese *(Alopochen aegyptiaca)*	213	213	212	2 *	Durban
Fulvous Whistling Duck *(Dendrocygna bicolor)*	4	4	4	0	Durban
Hawaiian Geese *(Branta sandvicensis)*	8	8	8	0	Durban
Little stint *(Calidris minuta)*	5	5	5	0	Newcastle
Mandarin Wood Duck *(Aix galericulata)*	1	1	1	0	Durban
Mute Swan *(Cygnus olor)*	2	2	2	0	Durban
Hooded Plover *(Thinornis rubricollis)*	2	2	1	0	Durban
Red bill teal duck *(Anas erythrorhyncha)*	2	2	2	0	Newcastle
Spotted Dikkop *(Burhinus capensis)*	1	1	1	0	Durban
Spurwing Geese *(Plectropterus gambensis)*	7	7	7	0	Durban
Yellow Billed Duck *(Anas undulata)*	3	3	3	0	Newcastle (n = 1) Durban (n = 2)
Wood sandpiper *(Tringa glareola)*	3	3	3	0	Stanger
White Faced Whistling Duck *(Dendrocygna viduata)*	8	8	4	0	Durban
Three-banded Plover *(Charadrius tricollaris)*	4	4	4	0	Newcastle
Unrecorded species	3	3	3	0	Not recorded
Total	278	278	272	2	

* One cloacal and one oropharyngeal sample collected from two different healthy Egyptian geese.
